# Alternative 3′ UTRs direct localization of functionally diverse protein isoforms in neuronal compartments

**DOI:** 10.1093/nar/gky1270

**Published:** 2018-12-22

**Authors:** Camilla Ciolli Mattioli, Aviv Rom, Vedran Franke, Koshi Imami, Gerard Arrey, Mandy Terne, Andrew Woehler, Altuna Akalin, Igor Ulitsky, Marina Chekulaeva

**Affiliations:** 1 Non-coding RNAs and mechanisms of cytoplasmic gene regulation, Berlin Institute for Medical Systems Biology, Max Delbrück Center for Molecular Medicine, Berlin, Germany; 2 Weizmann Institute of Science, Rehovot, Israel; 3 BIMSB Bioinformatics platform, Max Delbrück Center for Molecular Medicine, Berlin 13125, Germany; 4 Proteome Dynamics, Max Delbrück Center for Molecular Medicine, Berlin 13125, Germany; 5 Developmental Biology / Signal Transduction, Max Delbrück Center for Molecular Medicine, Berlin 13125, Germany; 6 BIMSB Light Microscopy platform, Max Delbrück Center for Molecular Medicine, Berlin 13125, Germany

## Abstract

The proper subcellular localization of RNAs and local translational regulation is crucial in highly compartmentalized cells, such as neurons. RNA localization is mediated by specific *cis-*regulatory elements usually found in mRNA 3′UTRs. Therefore, processes that generate alternative 3′UTRs—alternative splicing and polyadenylation—have the potential to diversify mRNA localization patterns in neurons. Here, we performed mapping of alternative 3′UTRs in neurites and soma isolated from mESC-derived neurons. Our analysis identified 593 genes with differentially localized 3′UTR isoforms. In particular, we have shown that two isoforms of *Cdc42* gene with distinct functions in neuronal polarity are differentially localized between neurites and soma of mESC-derived and mouse primary cortical neurons, at both mRNA and protein level. Using reporter assays and 3′UTR swapping experiments, we have identified the role of alternative 3′UTRs and mRNA transport in differential localization of alternative CDC42 protein isoforms. Moreover, we used SILAC to identify isoform-specific *Cdc42* 3′UTR-bound proteome with potential role in *Cdc42* localization and translation. Our analysis points to usage of alternative 3′UTR isoforms as a novel mechanism to provide for differential localization of functionally diverse alternative protein isoforms.

## INTRODUCTION

The neuron is a highly polarized cell, consisting of cell body (soma) and extensions (neurites - axons and dendrites). Such polarity is crucial to neuronal function and relies largely on asymmetric subcellular localization and translation of mRNAs (reviewed in (1)). mRNA localization and local regulation of translation allow neurons to control gene expression locally and thereby rapidly respond to local external stimuli. They have been implicated in multiple neuronal processes, including dendritic arborization, axon guidance and long-lasting changes in synaptic efficacy, which serve as a foundation of learning and memory. Recent high-throughput analyses demonstrated that hundreds to thousands of mRNAs localize to neurites (2–8). Moreover, our recent work showed that up to a half of local neuronal proteome can be explained by mRNA localization (7).

mRNA localization and translational control are often conferred by specific *cis-*regulatory elements located in 3′UTRs. Therefore, processes that generate alternative 3′UTRs—alternative splicing and polyadenylation—also affect mRNA localization (5,8). At least half of human genes generate alternative 3′UTRs by using alternative cleavage and polyadenylation sites (reviewed in (9)). Thus, alternative polyadenytion (APA) isoforms share a portion of their sequence. Brain-derived neurotrophic factor (BDNF) represents an example of such multi-UTR genes showing differential subcellular localization depending the 3′UTR length: BDNF with short 3′UTR is restricted to soma, while its long version is also localized in dendrites (10). Alternative splicing and, in particular, alternative last exon (ALE) usage usually generates isoforms that share most of the coding sequence, except for the very C-terminal part, but have completely distinct 3′UTRs. Indeed, the last splice junction in mRNA is normally found upstream of the stop codon, to escape the nonsense-mediated decay (NMD) (11).

One example of genes with ALE isoforms is *Cdc42* (cell division cycle 42). *Cdc42* is a small GTPase of the Rho family that shapes cellular morphology by controlling actin cytoskeleton (12–14). In the brain, *Cdc42* regulates axon and dendrite outgrowth, dendritic arborization and spine formation (15–20). Genetic ablation of *Cdc42* in brain resulted in disrupted cytoskeletal organization and enlargement of the growth cones (17). Alternative splicing of *Cdc42* generates two isoforms that differ in their last exon: exon 6 (E6) isoform is brain-specific and exon 7 (E7) isoform is ubiquitously expressed (18–20). The two isoforms have different 3′UTRs, but share most of their coding sequence, except for the C-terminal part encoding the last 10 amino acids, which are isoform-specific. These C-terminal sequences carry motifs that mediate differential post-translational modifications of the protein isoforms: E7 isoform is prenylated (CDC42E7 or CDC42-prenyl), while E6 is both prenylated and palmitoylated (CDC42E6 or CDC42-palm) (18,19,21). Moreover, the two protein isoforms were reported to have distinct functions and localization in neurons. CDC42E6 protein was found localized to dendritic spines and shown to play a role in their formation (18,20). CDC42E7 protein, on the other hand, functions in axonogenesis (20). However, it remains poorly understood how the two CDC42 protein isoforms achieve their differential localization and thereby perform different functions in neuronal growth and differentiation.

Here, we performed mapping of alternative 3′UTR isoforms in the neurites and soma of neurons differentiated from mouse embryonic stem cells (mESC), using 3′ mRNA-seq, total RNA-seq, Ribo-seq and mass spectrometry analyses. We identified ∼20 000 different 3′UTRs, and pairs of UTRs assigned to 593 genes showed differential usage in neurites versus soma (log_2_FC neurites/soma > 1). Curiously, we found that isoforms of *Cdc42* are differentially localized between neurites and soma not only at the protein, but also at the mRNA level: E7 isoform is more abundant in neurites, while E6—in soma. Moreover, the two mRNA isoforms are also locally translated in neuronal compartments. Using reporter assays and 3′UTR swapping experiments in mESC-derived and mouse cortical neurons, we showed that localization and local translation of CDC42E7 protein requires the E7 3′UTR. We also used SILAC (Stable isotope labeling by amino acids in cell culture) to identify E6 and E7 3′UTR-bound proteins with potential role in localization and local translation of *Cdc42* transcripts. Thus, our work suggests a novel mechanism for functional polarization of neurons, involving differential localization of alternative and functionally diverse CDC42 protein isoforms via usage of alternative 3′UTR isoforms.

## MATERIALS AND METHODS

### 3′ mRNA-seq

3′ mRNA-seq was performed with QuantSeq 3′ mRNA-Seq kit (Lexogen 015) according to the manufacturer′s recommendations. 3′ mRNA-seq was done in biological triplicates (soma) or duplicates (neurites), using 260 ng of total RNA from neurites or soma of mESC-derived neurons per sample. Libraries were pooled and sequenced on Illumina NextSeq 500 system with a single-end 150-cycle run.

### mESC culture, differentiation and neurite/soma separation

Mouse embryonic stem cells with doxycycline-inducible ASCL1 cassette (ASCL1-mESC) were cultured, differentiated and separated on neurites and soma as previously described (7), with the following modifications. First, mESCs were grown in AK medium to allow formation of embryoid bodies (EB) for the total of 4 days instead of 1 day (7). After 2 days EBs were split 1:1 and ASCL1 expression was induced with 3 μg ml^-1^doxycycline. Second, instead of removing one compartment (soma or neurites) with cotton swabs and using the porous membrane for isolation of the remaining compartment, each membrane was used to prepare both soma and neurite samples. For that, soma was detached by intensive washes with PBS, spun at 900 rpm for 3 min at 4°C and used for RNA or protein isolation. For neurites isolation, the remaining soma was removed from the top of the membrane with cotton swabs, the membrane with the neurites on the bottom was detached from the inlay and used for protein or RNA isolation. For protein extraction, neurites and soma were lyzed in 8 M UREA, 0.1 M Tris–HCl pH 7.5. For RNA isolation, TRIFast reagent (Peqlab) was used. Efficiency of separation was confirmed by somatic enrichment of histone H3 in western blot, with TUBB3 as a loading control.

### Primary cortical cultures

Primary mouse cortical neuron cultures were isolated from P0 mouse pups and cultured as previously described (22). For neurite/soma separation, neurons were plated at a density of 1 × 10^5^ cells/cm^2^ on double-coated (poly-d-lysine and laminin) cell inserts (Millicell 6-well PISP30R48, Millipore). At DIV21 cells were used either for imaging or for neurites and soma isolation. Isolation of subcellular compartments was performed as for mESC-derived neurons.

### RT and qPCR analysis

RNA from soma and neurites was treated with RQ1 DNase I to remove traces of gDNA and reverse-transcribed using the Maxima first strand cDNA synthesis kit (Thermo Fisher). qPCR was performed with sensiFAST SYBR No ROX qPCR kit (Bioline) with the primers provided in Supplementary Table S1. RT-qPCR reactions were carried out in technical duplicates and biological triplicates (soma) or duplicates (neurites), using a CFX96 Real-Time PCR system (Biorad). Relative neurites/soma expression levels were calculated using ΔΔC_t_ method, with *Capdh* as a reference RNA.

### Single molecule fluorescent in situ hybridization (smFISH)

smFISH was performed with *Cdc42* isoform-specific Stellaris probes sets (Biosearch Technologies) on primary mouse cortical neurons (P0, DIV18) according to the manufacturer′s instructions with few modifications. Instead of Vectashield medium, coverslips were mounted with a home-made antifade mounting medium: GLOX buffer supplemented with 37μg/ml glucose oxidase (G0543 Sigma) and 100 μg/ml catalase (C3155 Sigma). *Cdc42* isoforms expression was examined with 125 nM Quasar-labeled probes: Q570 for *Cdc42E6* and Q670 for *Cdc42E7*. Images were acquired with a Keyence microscope using a 60× oil objective. Maximum projections were performed using Fiji (ImageJ) using 10 slices with a Z-step of 0.3 mm. Images were further analyzed for signal quantification with StarSearch (Raj lab).

### DNA constructs

All the plasmids generated in this study and their sequences are available at the Addgene. *Cdc42E7* CDS and 3′UTR were PCR-amplified from cDNA prepared from mESC-derived neurons and fused with mCherry CDS at the C-terminus with the overlap PCR (mChe-Cdc42E7). For the 3′UTR swap experiment, mCherry-Cdc42E7 CDS was fused E6 3′UTRs using overlap PCR (mChe-Cdc42E7-E63′UTR). Next, mChe*-*Cdc42 reporters were cloned into lentiviral vectors. For expression in mESC-derived neurons, we used S2F-IMCg lentiviral vector with doxycycline-inducible CMV promoter (23), to simultaneously induce neuronal differentiation and expression of the reporters with doxycycline (Addgene 118614 and 118615). For expression in primary cortical cells, lentiviral vector with the synapsin promoter (Rusty Lansford, Addgene 51004) was used as a backbone for cloning of Che-*Cdc42* fusions (Addgene 118620 and 118622).

To generate RNA for GRNA chromatography (Cdc42E7 and E6 3′UTRs fused with boxB sites), the following plasmids were created to serve as templates for T3 in vitro transcription. E7 and E6 3′UTRs were PCR amplified and cloned into pBS-FLuc-5xboxB (24) to substitute FLuc, resulting in Cdc42E7-boxB (Addgene 118612) and Cdc42E6-boxB (Addgene 118609) plasmids. All constructs were confirmed by sequencing.

### Lentiviral transduction

To produce lentiviral particles, HEK293T cells were transfected in 10 cm dishes with 10 μg of the envelope (Addgene 12259), packaging (Addgene 12260) and transfer plasmids (mChe-Cdc42E7 and mChe-Cdc42E7-E63′UTR) in the ratio 1:1:2, using PEI. Next day after transfection, the medium was exchanged to 10 ml of DMEM with 2% FBS. Lentivirus-containing medium was harvested 72 hours post-transfection, and cleared from cell debris by centrifugation 5 min at 500 g. For transduction of mESC-derived neurons, 1 ml of supernatant was applied to 10^6^ cells plated on the microporous membrane. For primary neurons transduction, viral particles were concentrated using Lenti-X Concentrator (Clontech/TaKaRa) as recommended by the supplier and resuspended in 100 μl PBS. 3 μl of the concentrated virus was applied at 0.5 × 10^6^ of primary cortical neurons on the day of plating after the cells have attached to the membrane support.

### Western blotting

For western blotting, 5 μg of total protein from either neurites or soma was separated on a 12.5% Laemmli PAAG, and proteins were transferred to the PVDF membrane. The membrane was probed with the following primary antibodies: rabbit anti-mCherry antibody 1:5000 (167453 Abcam), rabbit anti-Tuj1/TUBB3 1:5000 (T2200 Sigma), rabbit anti-Histone H3 1:5000 (ab1791 Abcam), rabbit anti-Homer 1:1000 (160003 Synaptic System). mChe-CDC42E7 levels were quantified with Fiji, normalized to the levels of TUBB3 and presented as the neurites/soma ratios.

### Immunofluorescence and image analysis

Puro-PLA was performed as previously described (25). Briefly, mESC-derived neurons were incubated with 1 mg/ml puromycin for 5 minutes before fixation. After fixation in 4% paraformaldehyde, cells were immunostained with mouse anti-puromycin 1:2000 (3RH11 Kerafast) and rabbit anti-mCherry antibody 1:500 (167453 Abcam) using Duolink reagent (DUO92008 Sigma) according to the manufacture′s recommendations. To visualize neurites, cells were immunostained with chicken anti-neurofilament antibody 1:5000 (822601 Biolegend). Slides were mounted in Prolong gold antifade reagent with DAPI (Invitrogen). Images were acquired with a Leica TCS SP8 confocal microscope using ×63 oil objective. Puro-PLA signals were quantified using Fiji (ImageJ) function Analyze Particles. The function was applied to either soma, visualized by DAPI, or neurites, visualized by neurofilament staining at least 20 μm away from DAPI signal. Each analyzed region corresponded to 150 μm^2^ (∼ cell body size). The results represent the average from nine neurons expressing Cdc42E7 and 11 neurons expressing Cdc42E7-E6 3′UTR. The data are presented as the ratios of signals detected in neurites versus soma.

For immunostaining of primary neurons on the membrane, cells were fixed with 4% PFA for 10 min, permeabilized with 0.2% Triton X-100 in PBS for 10 min, and blocked with 1:5 dilution of the Western blocking reagent (11921673001 Sigma) in PBS for 30 min. Cells were probed with chicken anti-neurofilament antibody 1:10 000 (822601 Biolegend, ON at 4°C), washed with PBS-tween 0.05%, and incubated with fluorophore-coupled secondary antibodies for 1 h. Slides were mounted with ProLong Gold with DAPI (Cell Signaling). Images of cells growing on a porous insert were acquired on SP5 confocal microscope with a 40× oil objective and a pinhole of 90 μm as Z-stacks with 1024 × 1024 pixels *xy* resolution through the entire thickness of the cells and insert.

### GRNA chromatography and SILAC

boxB-Cdc42E7 and boxB-Cdc42E6 RNAs were generated using a T3 Megascript *in vitro* transcription kit (Thermo AM1338) according to the manufacturer′s recommendations. The template plasmids were linearized with HindIII. Post-synthesis, plasmid DNA was removed with DNAse I and RNA purified using Agencourt RNAClean XP beads (Beckman Coulter).

To prepare heavy (H) and light (L) lysates from mESC-derived neurons for SILAC experiments (26), mESCs were grown in light (L) or heavy (H: Arg +10 Da, Lys +8 Da) SILAC 80% 2i/20% mESC medium for six passages to ensure complete proteome labeling (>97%). Labeled mESCs were further differentiated into neurons in SILAC-customized differentiation media (L or H).

GRNA chromatography (27) was performed as described earlier (24) with some modifications. Per 60 μg of GST-lambda N fusion peptide, 20 μl of a 50% slurry of Glutathione-Sepharose 4B (Amersham, 17075601) in binding buffer (BB: 20 mM Tris–HCl pH 7.5, 200 mM NaCl, 1.5 mM MgCl_2_, 9% glycerol, 0.05% NP-40, 12 mg/ml heparin) were incubated on an orbital rocker for 30 min at room temperature. Glutathione-Sepharose beads were washed twice in 1 ml of BB to remove the unbound GST-lambda N, and incubated with 18 pmol RNA (boxB-Cdc42E7 or boxB-Cdc42E6) in 200 μl BB for 1 h at 4°C. The beads were washed twice with 1 ml BB and incubated with protein lysate prepared from heavy (H) or light (L) mESC-induced neurons (2.9 mg total protein, lysis buffer: 50 mM Tris–HCl pH 7.5, 150 mM KCl, 0.5% Triton, 0.4 mM Pefabloc) for 2 h at 4°C. The samples were pooled together according to the scheme in Figure 4A (H Cdc42E7 + L Cdc42E6 for fw and L Cdc42E7+ H Cdc42E6 for rev experiment), washed three times with 1 ml BB, and bound proteins were eluted with 0.15 μg RNAse A in 60 μl BB for 30 min at 30°C orbital shaker. Eluates were supplemented with 70 μl 2.5 M NaOAC pH 5.0, 1 μl Glycoblue (Ambion) and absolute EtOH up to 2 ml and incubated at 4°C overnight. Proteins were recovered by centrifugation at 18 000 g at 4°C for 30 min and subjected to liquid chromatography–tandem mass spectrometry (LC–MS/MS). LC–MS/MS and SILAC-based protein quantification were done as previously described (7). In short, LC–MS/MS analysis was performed with in-solution digested protein samples on a Q Exactive plus mass spectrometer (Thermo Scientific) according to (28). The averages of H/L (fw) and L/H (rev) ratios were used to measure relative protein abundance in boxB-cdc42E7 versus boxB-cdc42E7 complexes.

### Bioinformatical data analysis

3′ mRNA-seq analysis was performed largely as previously described (7). Raw reads were trimmed to remove adapters and low quality bases with the BBduk2 trimmer (https://sourceforge.net/projects/bbmap/): k = 10 threads = 12 ktrim = r qtrim = r minlength = 100 minoverlap = 9 trimq = 25. Cleaned reads were subsequently aligned using the STAR aligner (29) on the mm10 version of the mouse genome with the following parameters: –outFilterMultimapNmax 10 –outFilterMismatchNoverLmax 0.05. To define 3′ mRNA-seq clusters, all sequencing samples were merged. Genomic positions covered with 3′ most nucleotide of 3′ mRNA-seq reads were taken as putative cluster seeds. Positions containing less than three reads were disregarded. Clusters were obtained by merging all seed position within 25 bp. 3′ mRNA-seq cluster end (putative alternative polyadenylation site) was set as the position within the cluster with the highest read coverage, and the cluster width was set to 25 bp. To remove the effects of internal priming, 3′ mRNA-seq clusters completely contained within introns were removed from the analysis, as so were the clusters within 10 bp of borders of internal exons. A region from −45 bp to −35 bp upstream of the 3′ mRNA-seq cluster 3′ end was searched for known alternative polyadenylation motifs. The following motifs were used in the analysis: AATAAA, ATTAAA, TATAAA, AAGAAA, AGTAAA, AATATA, AATACA, CATAAA, GATAAA, TTTAAA. Motif was considered as found if there was a perfect match. A region from −8 bp to +10 bp around the 3′ mRNA-seq cluster 3′ end was search for the known polyadenylation signal. The following motif was used: AAAAAAAA, with two allowed mismatches. All downstream analysis was done with 3′ mRNA-seq clusters containing a valid APA, and PolyA motifs, which were located in annotated 3′UTRs. Pairs of UTRs with the difference in usage between soma and neurites were then computed as in (30). Briefly, for each gene we first counted the number of reads assigned to each 3′ end in each of the two samples (soma/neurites), and computed for each 3′ end its ′relative usage′ (number of reads mapping to the 3′ end/number of reads assigned to all the 3′ ends of that gene combined). Only 3′ ends with at least five reads in one of the libraries were considered. We then identified for each gene the 3′ end which showed the maximal ‘positive change’ in relative usage (arbitrarily defining usage in soma as positive) and the one wth maximal ‘negative change’; Genes for which one of those changes was at least 20% were considered further, and the pair of UTRs with the maximal positive and negative changes were considered the ‘switching’ isoforms. To calculate translation efficiencies (TEs) of *Cdc42* isoforms in neurites and soma, we counted reads overlapping with the coding parts of E6 and E7 in RNA-seq and Ribo-seq datasets, normalized them to the number of reads mapping to the all coding sequences annotated in RefSeq, and divided resulting Ribo-seq reads by RNA-seq reads.

## RESULTS

### Identification of differentially localized 3′UTRs

**Figure 1.**
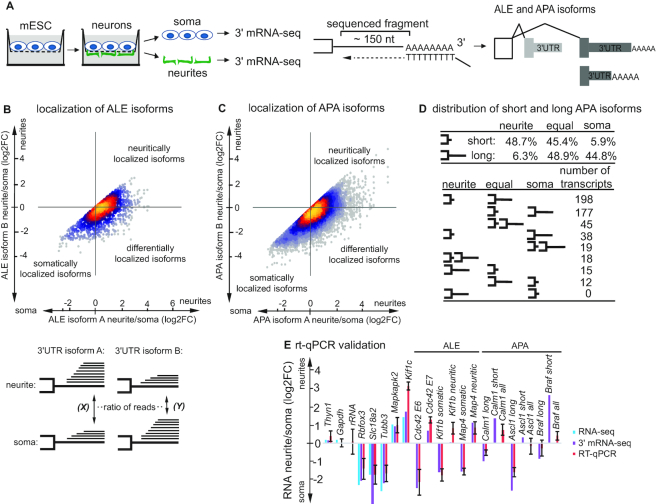
Alternative polyadenylation sites usage and resulting alternative 3′UTRs affect mRNA localization to neurites. (**A**) Scheme of neurite/soma separation and 3′ mRNA-seq. mESC-derived neurons are grown on a microporous membrane so that neurites extend on the lower side of the membrane to enable separation of the soma and neurites. RNA isolated from subcellular compartments is subjected to 3′ mRNA-seq, which is based on oligodT priming and enables amplification and sequencing of polyadenylated mRNA 3′ ends. The resulting data are analyzed for the ALE and APA isoforms. (**B**) Scatterplot illustrating differential localization patterns for transcripts with ALE 3′UTR isoforms. 3′UTR isoforms are designated as A and B on the condition that A is equally or more represented in neurites than B (see the scheme below the plot). Enrichment in neurites for isoform A (X) is plotted against the same enrichment for isoform B (Y). Dots falling on diagonal correspond to genes with similar localization patterns of alternative 3′UTR isoforms. For the rest of the genes, transcripts with alternative 3′UTRs show differential localization between neurites and soma. Coloring indicates local point density. (**C**) Plot illustrating differential localization patterns for transcripts with APA 3′UTR isoforms. Data are presented as in (B). (**D**) Distribution of short and long APA isoforms between neurites and soma. The top panel shows percentages of short (]-) and long (]*—*) APA isoforms, which are enriched in neurites, enriched in soma, or else equally distributed. The lower panel shows localization patterns of short and long APA isoforms deriving from the same gene. Neurites: enriched in neurites >2-fold; equal: <2-fold change between neurites and soma; soma: enriched in soma >2-fold. (**E**) qRT-PCR for selected differentially localized alternative 3′UTRs. Error bars represent SD for two (neurites) to three (soma) biological replicates. *Gapdh* (reference RNA), *Thyn1*, rRNA were used as unlocalized controls, Rbfox3, Slc18a2, Tubb3 as soma-localized, *Mapkap2, Kif1c* as neurite-localized controls. ENSEMBL identifiers of the isoforms: *Cdc42E6* ENSMUST00000030417.9; *Cdc42E7* ENSMUST00000051477.12; *Kif1b* somatic ENSMUST00000060537.12; *Kif1b* neuritic ENSMUST00000030806.5; Map4 somatic ENSMUST00000169851.7; Map4 neuritic ENSMUST00000035055.14. For a reference, we also show neurite/soma enrichment based on 3′ mRNA-seq (purple bars) and RNA-seq data (blue bars, not isoform-specific) next to the qRT-PCR data (red bars). In case of APA isoforms, the qRT-PCR data are shown for a long isoform and both isoforms combined (all), as short APA isoforms cannot be distinguished from long APA isoforms by qPCR.

To identify differentially localized mRNA isoforms, we isolated neuronal cell compartments—soma and neurites—for further 3′ mRNA-seq analysis (Figure 1A). For that, we cultured neurons on a microporous membrane so that soma stay on the top and neurites grow through the pores the lower side of the membrane, as it was done in our previous work (7). As a test system, we used excitatory neurons differentiated from mouse embryonic stem cells (mESC) by inducible expression of a pioneer proneural transcription factor ASCL1 (7,31–34). As ASCL1 is expressed in every cell, this procotol generates a highly homogenous population of neurons (35) and is particularly well suited for genome-wide analyses. Resulting neurons possess all basic neuronal properties, i.e. express mature neuronal markers, exhibit typical passive and active intrinsic membrane properties, and form functional synapses (7,32,36,37,38).

To study how alternative splicing and polyadenylation affect mRNA localization on transcriptome-wide level, we performed 3′ mRNA-seq of neurites and soma isolated from mESC-derived neurons. 3′ mRNA-seq allows mapping and quantification of mRNA 3′ ends (Figure 1A). Using 22 327 clusters of 3′ mRNA-seq reads we annotated 19 175 different 3′UTRs for 10,868 genes (Supplementary Table S2). For 4149 genes we annotated multiple 3′ ends, and for each of those focused on the pair of ends which showed the highest difference in usage between soma and neurites. For 3,675 genes this change corresponded to APA isoforms, and for 474 to ALE (Figure 1B and C). As expected, alternative 3′ ends corresponding to APA events were more concordant in their soma/neurite ratios (intraclass correlation 0.462) than those corresponding to ALE (intraclass correlation 0.324). We then further focused on 593 genes for which the relative change in usage for the most variable pair of alternative UTRs (522 APA and 71 ALE) was at least 20%. For 303 of these genes the most commonly used 3′ end differed between soma and neurites.

Strikingly, for 534/593 genes (90%) the 3′ end corresponding to the more proximal cleavage and polyadenylation site (short APA isoforms) was preferentially localized in the neurites. The 3′UTR of the neurite-enriched isoforms were shorted in 59.2% of the ALE pairs, and 92.3% of the APA pairs. The differences in the induced UTR lengths were substantial—the average 3′UTR length of the soma-enriched isoforms was 2398 nt compared to 1080 nt for the neurite-enriched isoforms. Further, when we considered the neurites/soma ratio of 3′ mRNA-seq reads mapping to each APA isoform, 48.7% of short isoforms and only 6.3% of the long ones were found enriched >2-fold in neurites (Figure 1D). We next examined nine possible combinations of localization patterns (neurite-enriched > 2-fold, soma-enriched > 2-fold, and all others—‘equal’) for two alternative APA isoforms (short and long). Consistently with the general trend, the most abundant combination (198 out of 522) was represented by transcript pairs where short isoforms were enriched in neurites and long were equally distributed (Figure 4D). Transcripts with equally distributed short APA isoform and somatically enriched long APA isoforms were also quite common (177 out of 522).

We validated differential localization patterns for selected candidates with alternative 3′UTRs by qPCR on isolated neurites and soma (Figure 1D). Localization ratios measured by 3′ mRNA-seq and by qRT-PCR were highly consistent (Pearson *r* = 0.866, Supplementary Figure S1). Among genes with differentially localized ALE isoforms we detected *Kif1b*, a kinesin-3 family anterograde motor protein implicated in the axonal transport of mitochondria and synaptic vesicles (39,40). Consistently, mutations in the motor domain of *Kif1b* were found in one of the motor and sensory neuropathies, Charcot-Marie-Tooth disease (40). The two asymmetrically localized isoforms of *Kif1b* Isoforms differ in the cargo-binding C-terminal domain and therefore are expected to bind and localize distinct cargos. Other examples of differentially localized isoforms are represented by a microtubule-associated protein 4 (*Map4*), contributing to the microtubule-dependent transport in neurons (41), and *Cdc42*, involved in neuronal polarity (12–14).

### Isoforms of *Cdc42* are differentially localized between neurites and soma at both mRNA and protein level

**Figure 2.**
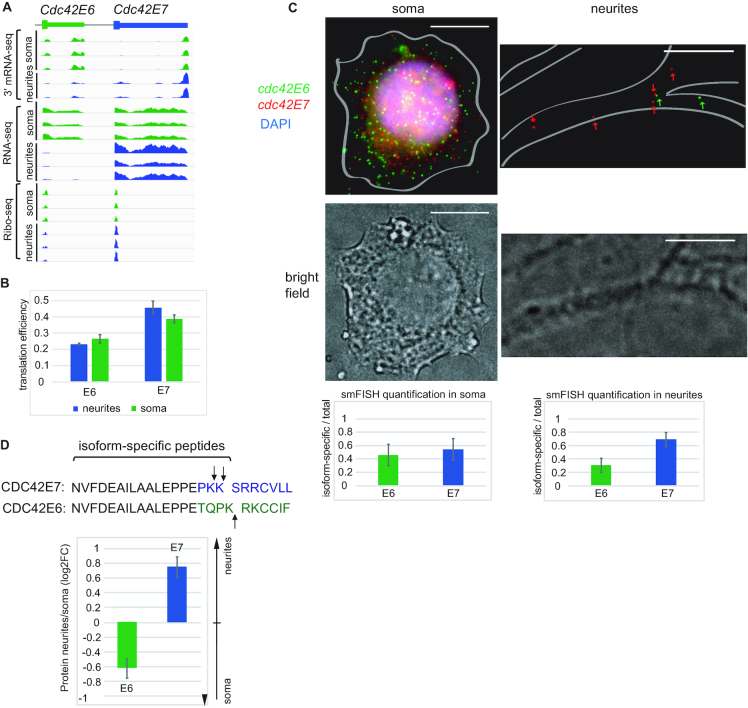
Isoforms of the cell polarity gene *Cdc42* are differentially localized between soma and neurites at mRNA and protein levels. (**A**) The snapshops from the genome browser showing distribution of *Cdc42* sequencing reads (3′ mRNA-seq, total RNA-seq, Ribo-seq) between neurites and soma of mESC-derived neurons. Total RNA-seq and Ribo-seq data were reported in our previous work (7). (**B**) Translation efficiencies (TEs) of *Cdc42* isoforms in neurites and soma. See Materials and Methods for details. Errors bars are SEM. Green bars: TE in soma, blue bars: TE in neurites. (**C**) smFISH of *Cdc42*E6 and *Cdc42*E7 transcripts with isoform-specific Stellaris probes was performed in mouse cortical neurons from P0 at DIV18 *Cdc42*E6: green (Q570), *Cdc42*E7: red (Q670), DAPI: blue, scale bar: 10 μm. Cell borders are outlined based on the brightfield image. Fluorescent spots corresponding to E6 and E7 isoforms were counted using StarSearch (Raj lab) and the quantification plots presented below the images (left: soma, right: neurites). The Y-axis shows ratios of isoform-specific to total *Cdc42* signals in the analyzed subcellular compartment. The data represent averages of 13 neurons and the error bars are SD. (**D**) CDC42 protein isoforms are differentially distributed between neurites and soma. CDC42 isoform-specific peptides were extracted from the mass spectrometry data generated in our previous work (7) and used to evaluate relative levels of E6 and E7 isoforms in neurites and soma of mESC-derived neurons. The sequences show 25 C-terminal amino acids from which isoform-specific peptides are derived, with arrows pointing at the positions of trypsin digest. Values were normalized to the intensities of peptides shared between E6 and E7 isoforms. Error bars represent SD for 3 biological replicates.

We next focused on the cell polarity gene *Cdc42* (Figure 2). *Cdc42* is involved in cytoskeletal organization (reviewed in (42)). It uses actin cytoskeletal regulation to reshape cellular morphology, inducing filopodia in many cell types, and in neurons, it has been implicated in inducing axon and dendrite outgrowth. The *Cdc42* isoform containing alternative exon 6 (*Cdc42E6*, brain-specific) is mostly somatic, while the exon 7 isoform (*Cdc42E7*, ubiquitous) is enriched in neurites, both in our 3′ mRNA-seq and previously generated total RNA-seq data (7) (Figure 2A).

Moreover, our Ribo-seq analysis of neurites and soma showed that differentially localized *Cdc42* isoforms are also locally translated in subcellular compartments (Figure 2A). We next evaluated translation efficiencies (TEs), i.e. the ratios of ribosome footprints to mRNA fragments, of *Cdc42* isoforms in neurites and soma (Figure 2B). This analysis showed that neuritically enriched E7 isoform is also translated with slightly higher efficiency in neurites, while somatically enriched E6—in soma. Noteworthy, for both isoforms the differences in TE between neurites and soma were not as profound as differences in mRNA levels (Figure 2A, Supplementary Table S2), suggesting that most regulation is via differential localization of mRNA isoforms.

We wondered if differential localization of *Cdc42* isoforms is also happening in primary neurons. To test that, we analyzed localization of *Cdc42E6 and Cdc42E7* transcripts in mouse cortical neurons using single molecule fluorescent in situ hybridization (smFISH, Figure 2C). Quantification of signal produced by isoform-specific Stellaris probes confirmed that *Cdc42E7* is indeed more abundant in outgrowths of cortical neurons than *Cdc42E6* isoform, similarly to mESC-derived neurons.

The two *Cdc42* splicing isoforms share most of the protein sequence, but differ in the very C-terminus of the CDS and in 3′UTR (Figure 2D). Given differential localization and translation of *Cdc42* isoforms, we decided to examine if protein isoforms are also differentially localized. For that, we reanalyzed liquid chromatography–tandem mass spectrometry (LC–MS/MS) data on neurites and soma generated in our previous work (7). We compared the intensities of the peptides specific to either E6 or E7 isoforms (Figure 2D). Indeed, we found that CDC42E7 protein is more abundant in neurites, and CDC42E6—in soma. Interestingly, the two protein isoforms are reported to have different functions in hippocampal neurons: the CDC42E6 is required for normal development of dendritic spines, whereas the CDC42E7 is involved in axonogenesis (18,20). Altering the ratio of *Cdc42* isoforms leads to increased anxiety in mice (20).

### Location of CDC42E7 protein to neurites requires the E7 3′ UTR

**Figure 3.**
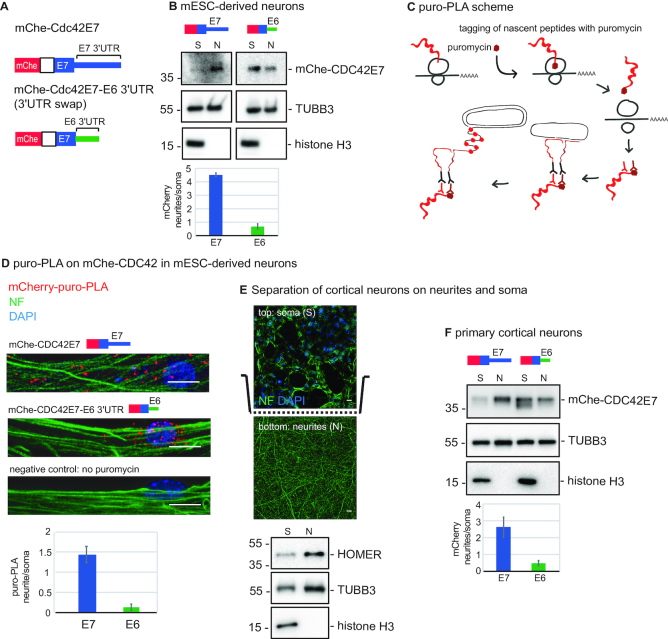
Differential localization of CDC42 isoforms requires alternative *Cdc42* 3′UTRs. (**A**) The scheme of *Cdc42* reporters used in this study: mCherry was fused to the CDS of *Cdc42*E7 with either E7 3′UTR (E7 construct), or with E6 3′UTR (swap, or E6 construct). **(B)** Western blotting showing localization of mChe-CDC42E7 protein in mESC-derived neurons infected with either E7 or E6 3′UTR reporter, and separated on neurites (N) and soma (S) using the scheme in Figure 1A. Histone H3 was used as a soma marker and TUBB3 as a loading control. Intensity of the mChe-CDC42E7 signal was quantified, normalized to TUBB3, and presented below the blot as fold enrichment in neurites versus soma. (**C**) Scheme showing the working principle of puro-PLA assay used to detect de novo synthesized proteins. (**D**) Puro-PLA images showing localized translation of CDC42E7 from either E7 or E6 3′UTR reporter-infected mESC-derived neurons. For a negative control, puromycin treatment was omitted on mChe-CDC42E7 sample. mChe-CDC42E7-puro-PLA: red, Neurofilament (NF): green, DAPI: blue, scale bar: 10 μm. Quantification of the relative puro-PLA signals (CDC42E6/total *and* CDC42E7/total) is shown below the images. The error bars represent SD for 9 (Cdc42E7) and 11 (Cdc42E7-E6 3′UTR) neurons. (**E**) Separation of mouse cortical neurons on soma and neurites using microporous membrane as illustrated in Figure 1A. Efficiency of separation is confirmed with fluorescent micrographs of the primary cortical neurons above (top) and below the membrane (bottom). Neurofilament (NF): green, DAPI: blue, scale bar: 10 μm. It is to be expected that the initial part of neurites can be seen of top of the membrane. Additionally, isolated neurites and soma preparations were analyzed by western blot for somatic (histone H3) and neuritic (HOMER1) markers. The component of neuronal microtubules TUBB3 was used as a loading control. (**F**) Western blotting showing localization of mChe-CDC42E7 protein in primary cortical neurons infected with either E7 or E6 3′UTR reporter, and separated on neurites and soma as shown in (**E**). Histone H3 was used as a soma marker and TUBB3 as a loading control.

The question arises how the two CDC42 protein isoforms localize to two different subcellular compartments. Prior studies implied the role of differential protein modifications in this process (18,19,21). However our 3′ mRNA-seq, total RNA-seq and Ribo-seq data (Figure 2A) suggest a role of alternative 3′UTRs and mRNA transport in this process. As no antibodies specifically and efficiently recognizing CDC42 isoforms are available, we generated a reporter construct expressing mCherry-tagged CDC42E7 (mChe-CDC42E7) to test this hypothesis (Figure 3A). We transfected mChe-Cdc42E7 into mESCs and differentiated them into neurons. To test if the reporter fatefully recapitulates behavior of the endogenous CDC42E7 protein, we applied neurites/soma separation scheme and analyzed distribution of mChe-CDC42E7 protein between neurites and soma by western blotting (Figure 3B). To ensure the efficiency of separation we used the nuclear marker histone H3, which was detected only in the soma preparation. As expected, the protein product of the mChe-Cdc42E7 reporter showed preferential localization to neurites. To test if this localization is mediated by E7 3′UTR, we swapped the E7 3′UTR for E6 3′UTR (mChe-Cdc42E7-E63′UTR), and repeated the experiment. Strikingly, mChe-CDC42E7 protein produced from E6 3′UTR reporter showed stronger localization to soma. These results point to the role of alternative 3′UTRs, rather than differences in protein sequence, as the defining factor in localization of CDC42 protein isoforms.

As our data show that the Cdc42E7 isoform is not only localized to neurites, but also locally translated (Figure 2A), we decided to test if its neuritic translation is dependent on E7 3′UTR. We visualized *de novo* synthesis of mChe-CDC42E7 protein from either E7 3′UTR- and E6 3′UTR-containting reporters, using constructs described above and the puromycin proximity ligation assay (puro-PLA), which combines puromycin-tagging of newly synthesized proteins with the proximity-ligation assay (25) (Figure 3C). Puromycin becomes incorporated into the nascent polypeptide chains on ribosomes due to its structural analogy with the aminoacylated 3′-end of tRNA. Puromycin-tagged mChe-CDC42E7 is then detected with the proximity-ligation assay with two antibodies: an anti-puromycin antibody that binds *de novo*-produced proteins and an anti-mCherry antibody recognizing mChe-CDC42E7. The secondary antibodies are coupled to different oligonucleotide probes, and only when the two probes occur in close proximity can the linker oligonucleotide hybridize to both for rolling circle amplification. The amplified sequences are detected by *in situ* hybridization.

Using puro-PLA, we visualized mChe-CDC42E7 protein, newly synthesized from either mChe-Cdc42E7 or mChe-Cdc42E7-E63′UTR reporter (Figure 3D). We observed that reporter with E7 3′UTR is mainly translated in neurites, while swapping it for E6 3′UTR leads to more abundant translation in soma. No puro-PLA signal was detected in the sample, for which puromycin treatment was omitted, confirming signal specificity (Figure 3D, no puromycin). These data suggest that isoform-specific 3′UTRs mediate not only localization, but also local translation of CDC42 protein.

Next, we decided to test if alternative 3′UTRs also determine localization of CDC42 protein isoforms in primary cortical neurons. For that, we infected mouse cortical neurons with mChe-Cdc42E7 and mChe-Cdc42E7-E63′UTR lentiviral constructs and separated them to neurites and soma using porous membrane support, as it was described for mESC-derived neurons (7). The coating agent on the lower side of the membrane provided cues to stimulate neurite growth through the pores on the lower side of the membrane. We tested the efficiency of separation by immunostaining of primary cortical neurons on the porous membrane and western blotting of isolated neurites and soma fractions with nuclear and neurite markers. (Figure 3E). Indeed, histone H3 was detected only in soma and synaptic protein HOMER1 was enriched in neurites. Moreover, neurites visualized with neurofilament staining were found primarily on the lower side of the membrane while soma, visualized with DAPI, was detected on the top. We then analyzed isolated neurites and soma by western blotting against mChe-CDC42E7 (Figure 3F). We detected CDC42E7 protein enriched in neurites, when it was encoded by E7 3′UTR-containing mRNA, while swapping E7 3′UTR for E6 changed CDC42E7 protein localization to more somatic. This analysis showed that proper localization of CDC42E7 protein requires E7 3′UTR, also in primary cortical neurons.

### Identification of the isoform-specific interactome of the *Cdc42* 3′UTRs

**Figure 4.**
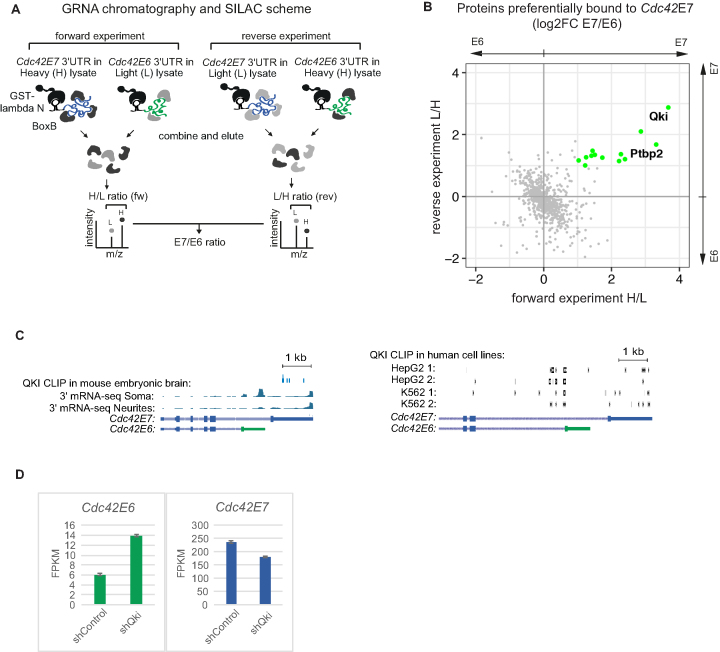
Identification of the proteins bound to the *Cdc42* 3′UTRs. (**A**) Scheme illustrating GRNA chromatography combined with SILAC (Stable isotope labeling with amino acids in cell culture) to identify proteins bound to E6 and E7 3′UTRs of *Cdc42*. boxB-Cdc42E7 3′UTR and boxB-Cdc42E6 3′UTR RNAs were incubated with lysate prepared from either light (L) or heavy (H) mESC-derived neurons. Bound proteins were eluted with RNAse A and combined as shown in the scheme (H E7 + L E6 in forward experiment and L E6 + H E7 in reverse experiment), and analyzed by mass spectrometry. H/L (forward) and L/H (reverse) ratios for each protein represent fold of enrichment of a given protein in complexes formed on E7 versus E6 *Cdc42* 3′UTR. (**B**) Scatterplot of E7/E6 protein ratios obtained in forward and reverse experiments. Green: proteins overexpressed in enriched in E7-bound complexes by more than 2-fold in both forward and reverse experiments. (**C**) QKI sites are found in *Cdc42* E7 3′UTR and upstream of E6 splice site, but not in E6 3′UTR. QKI CLIP data from mouse embryonic brain (44) and human HepG2 and K562 lines (two biological replicates, BioRxiv: https://doi.org/10.1101/179648) were re-analyzed to visualize QKI sites in *Cdc42*. The snapshops from the genome browser showing distribution of *Cdc42* 3′ mRNA-seq reads between neurites and soma and position of QKI CLIP reads on *Cdc42*. (**D**) Knockdown of QKI in mouse neural stem cells (NSCs) changes distribution of *Cdc42* splicing isoforms. We re-analyzed the mRNA-seq data produced from E14.5 mouse cortex cells infected with a lentivirus encoding Qki-targeting shRNA (shQki) and control no-target shRNA (shControl) (44). Expression data for *Cdc42* isoforms (E6 and E7) in control and Qki-depleted cells are presented as FPKM (fragments per kilobase per million reads).

mRNA localization is mediated by RNA-binding proteins (RBPs) that bind to mRNA and recruit components of transport machinery (reviewed in (1). Thus, having shown the role of *Cdc42* 3′UTRs in localization, we decided to look for RBPs that bind to these 3′UTRs and could mediate RNA transport and local translation. To identify interacting RBPs, we employed an RNA affinity capture approach, GRNA chromatography (27), combined with the mass spectrometry-based approach SILAC that detects differences in protein abundance between samples using non-radioactive isotopic labeling (26). We tagged the *Cdc42* E7 and E6 3′UTRs with five copies of boxB sequence, which binds lambda N peptide, and incubated resulting E7-boxB and E6-boxB RNAs with neuronal lysates. Lysates were produced from neurons labeled with either heavy (H) or light (L) amino acid isotopes. The complexes formed on E7-boxB RNA and E6-boxB RNA were isolated by binding to a lambda N-GST fusion protein immobilized on glutathione beads. We pooled differentially labeled protein samples together (H E7-boxB + L E6-boxB in forward experiment and H E6-boxB + L E7-boxB in reverse experiment, Figure 4A) and eluted proteins contained in the complexes with RNAse A for further proteomic analysis. The forward and reverse experiments represent ‘label swap’ replicates standardly used in SILAC to eliminate biases of the labeling procedure. The ratios of peak intensities, H/L in forward experiment and L/H in reverse experiment, quantify relative protein levels in E7 versus E6 complexes. Using this approach, we identified 13 RBPs enriched in E7 versus E6 complexes by at least 2-fold (Figure 4B, Supplementary Table S3). Interestingly, among identified *Cdc42E7*-bound RBPs is Quaking (QKI), previously suggested to play a role in RNA localization and translation in perisynaptic astrocyte processes (43), and polypyrimidine tract binding protein 2 (PTBP2), which is involved in *Cdc42* splicing (20).

To evaluate the role of these candidates in the metabolism of *Cdc42* isoforms, we analyzed available crosslinking and immunoprecipitation (CLIP) data. While PTBP2-binding sites were found on both *Cdc42* isoforms (data not shown), CLIP of QKI in embryonic mouse brain (44) suggested that it indeed binds E7, but not E6 isoform (Figure 4C, left panel). We next examined QKI CLIP dataset performed on non-neuronal human cells (BioRxiv: https://doi.org/10.1101/179648), where only E7 isoform is produced due to skipping of the brain-specific exon E6. This analysis identified an additional QKI-binding site upstream of the E6 splice site, suggestive of QKI role in splice site selection (Figure 4C, right panel). The absence of this site in mouse brain dataset (44) is likely due to different cell types (i.e. brain versus cancer cell lines), and vastly different depth (11,931 versus 51 927–261 971 CLIP clusters). Moreover, re-analysis the mRNA-seq data produced from QKI knockdown E14.5 mouse cortex cells (44) showed that depletion of QKI changed the ratio *Cdc42* isoforms, increasing the levels of E6 and decreasing the levels of E7 (Figure 4D). These data suggest the role of QKI in alternative splicing of *Cdc42* isoforms and/or stabilisation of E7 isoform.

## DISCUSSION

mRNA localization is mediated by *cis-*acting elements in mRNA itself, most often located in its 3′UTR. For example, a 54-nt element within *beta-actin* mRNA 3′UTR, called zipcode, binds zipcode binding protein 1 (ZBP1) and targets mRNA to the cell periphery (45–47). The AU-rich regulatory elements (AREs) in the 3′UTR of *Tau* and *GAP-43* mRNAs recruit the ELAV-like/Hu family protein HuD and localize mRNA to axons (48,49). Given the established role of 3′UTRs in mRNA transport, formation of alternative 3′UTR isoforms can provide a mechanism of creating diverse subcellular localization patterns for their protein products.

Alternative 3′UTR isoforms are generated via two mechanisms: (i) alternative polyadenytion, that results in a short and a long APA isoforms sharing a part of their 3′UTR, and (ii) alternative splicing of the last exon that generates ALE isoforms with completely different 3′UTR sequences. Based on that, ALE isoforms have higher chance to be differentially localized than APA isoforms. Indeed, our analysis identified 522/71 genes with substantially different localization of APA/ALE isoforms between neurite and soma. Unexpectedly, we find that short APA isoforms are preferentially localized to neurites (Figure 1D). Given that the sequence of short APA isoforms is contained within long APA isoforms, this result can be explained in several ways: (a) sequences present exclusively in long APA isoforms anchor them in soma and prevent their localization to neurites; (b) extra sequences present in the long APA isoforms mediate their stabilization in soma or degradation in neurites via localized trans-acting factors, e.g. RBPs and miRNAs; (c) sequences within shorter isoforms required for localization are hidden within secondary structures formed by long APA isoforms; (d) long APA isoforms undergo 3′UTR shortening in neurites (50,51). Interestingly, prior studies obtained conflicting results on localization of short and long APA isoforms. The study of Taliaferro *et al.* (5) reported similar numbers of short and long APA isoforms localized to neurites of CAD and N2A neuroblastoma cell lines. Analysis of rat brain slices detected slightly higher number of long APA isoforms in neuropil than in somata (8). Also, longer isoforms were reported in axons of cultured rat sympathetic neurons (BioRxiv: https://doi.org/10.1101/170100). Possible explanation for this discrepancy is usage of different test systems. In addition, brain slices contain not only neurons, but also glia, and computational subtraction of glial transcripts might bias quantification of localization (8).

**Figure 5.**
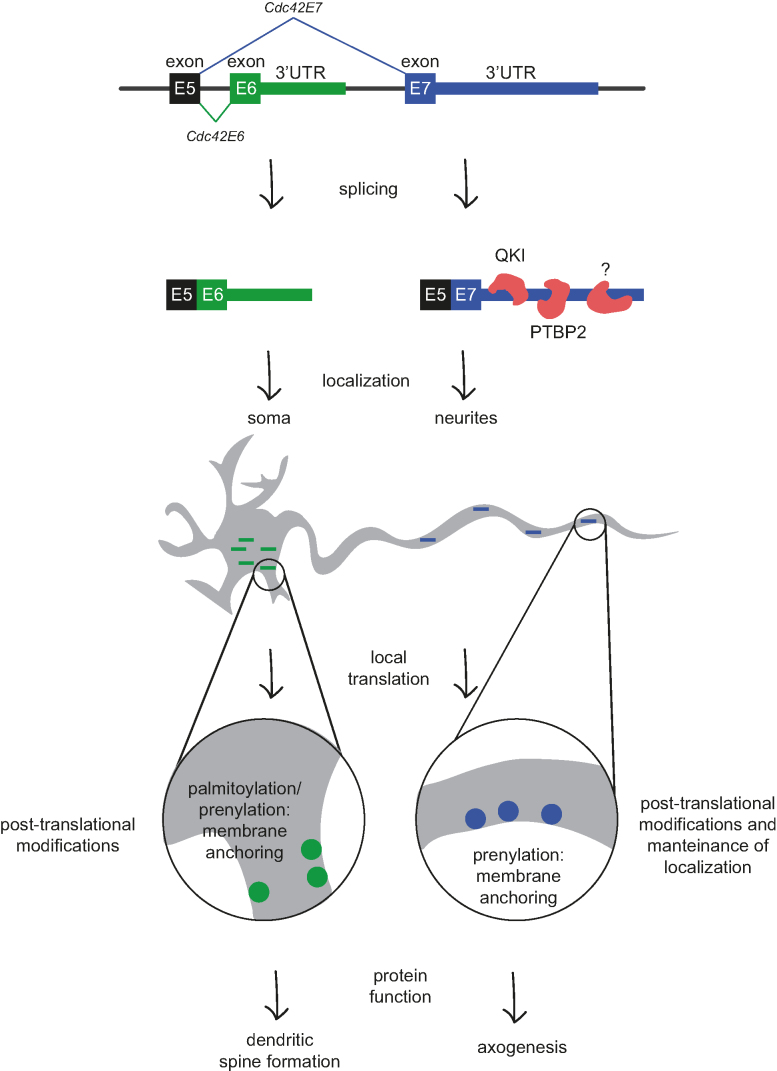
Speculative model illustrating the mechanism of CDC42 isoforms localization via alternative 3′ UTR usage. Neurons express two isoforms of *Cdc42*, characterized by a different last exon. Through their distinct 3′UTRs, the mRNA isoforms are differentially localized between soma and neurites, with *Cdc42E6* being soma-enriched and *Cdc42E7*—neurite-enriched. The asymmetric mRNA localization and their local translation lead to accumulation of protein isoforms in distinct subcellular compartments. Modifications that increase protein hydrophobicity tether them to the membrane and likely maintain their localization patterns. This isoform-specific regulation of mRNA localization enables CDC42 protein isoforms to function in distinct neuronal compartments.

Among differentially localized ALE isoforms we identified cell polarity gene *Cdc42* with known role in shaping neuron morphology (Figure 2A). Interestingly, CDC42 protein isoforms were reported to have functions in different neuronal compartment: CDC42E7 contributes to axonogenesis and CDC42E6 in formation of dendritic spines (15–20). However the mechanism behind differential localization of protein isoforms remained poorly investigated. As protein isoforms differ in their C-terminal ten amino acids, they can be differentially modified—either prenylated or palmitoylated (18,19,21). These modifications increase protein hydrophobicity and were suggested to tether proteins to the membrane, but could not explain differential protein localization between soma and neurites. Our comprehensive combination of datasets, including 3′ mRNA-seq, total RNA-seq, Ribo-seq and mass spectrometry analysis of neurites and soma, allowed us to investigate local expression of *Cdc42* at different levels. We found that *Cdc42* mRNA isoforms are differentially localized and their alternative 3′UTRs are required for proper localization of the protein isoforms. Our data thus point to the role of 3′UTRs and mRNA localization in generating differential localization of CDC42 protein isoforms (Figure 5). We show that protein modification motif is not sufficient to properly localize proteins, although it likely contributes to maintaining protein localization by anchoring the protein to the membrane and preventing diffusion. Importantly, this mechanism is functional not only in mESC-derived neurons, but also in primary cortical neurons. Consistently, we detected differential localization of *Cdc42* isoforms in previously reported 3′ mRNA-seq from rat somata and neuropil (8). Recent work of Twiss and colleagues (BioRxiv: http://dx.doi.org/10.1101/366369) also supports the role of *Cdc42* E7 localization in cultured sensory neurons.

It remains to be understood, why different *Cdc42* isoforms are required in different subcellular compartments. As protein isoforms are differentially modified, the nature of these modifications—prenylation and palmitoylation (18,19,21)—may contribute to different functionality of the proteins. For instance, although both modifications mediate interaction with the membrane, the dynamic of these interactions is likely to be different depending on modification. Given the role of CDC42 in regulation of actin cytoskeleton, the requirement for different CDC42 isoforms may also be mediated by the differences in local cytoskeleton. Indeed, prior studies showed that components of cytoskeleton - *β-actin* and *γ-actin*—are differentially localized between growth cones and soma of cultured cerebrocortical neurons (52).

Moreover, we identified 13 *Cdc42E7* 3′UTRs-bound RBPs. Among them is QKI, a STAR (signal transduction and activation of RNA) family of K homology (KH) domain-containing RBP with multiple functions in RNA metabolism, including splicing, mRNA stabilization, and miRNA and circRNA biogenesis (reviewed in (53)). Moreover, QKI was reported to contribute to RNA localization and translation in perisynaptic astrocyte processes (43). Available QKI high-throughput sequencing of RNA isolated by crosslinking immunoprecipitation (HITS-CLIP) data (44) indeed suggest presence of QKI-binding sites in the 3′UTRs of *Cdc42E7*, but not *Cdc42E6* (Figure 4C). Further, knockdown of QKI in neuronal stem cells leads to a significant decrease in the usage of the E7 isoform concomitant with increase in the usage of E6 (Figure 4D). PTBP2 is another RBP that bound to E7 3′UTRs more efficiently than to E6 3′UTRs. PTBP2 is a neuronal protein that regulates alternative splicing during neuronal development (54). In fact, PTBP1 and PTBP2 proteins are involved in alternative splicing *Cdc42* in neurons, stimulating production of E7 isoform (20). It is tempting to speculate that some of these proteins remain bound to the E7 isoform following mRNA maturation, and play a role in localization and translation of *Cdc42E7* isoform in neurites. Moreover, since *Cdc42*E7 is not neuron-specific, the localization mechanism for this isoform may not be limited to neurons. Another interesting possibility is that neuron-specific E6 isoform contains elements that restrict its localization to soma.

To sum up, our analysis identified hundreds of 3′UTR isoforms differentially localized between neuronal subcellular compartments, using 3′ mRNA-seq, total RNA-seq, Ribo-seq and mass spectrometry analyses. Moreover, we found that isoforms of CDC42 protein with distinct functions in neuronal polarity become localized to different subcellular compartments due to differential localization of mRNA isoforms encoding them. This finding suggests that usage of alternative 3′UTR isoforms can generate differential localization patterns for functionally diverse proteins and represents a novel mechanism for establishment of neuronal polarity.

## DATA AVAILABILITY

The NGS data reported in this paper are deposited on Array Express with the accession numbers E-MTAB-4978, E-MTAB-4979 and E-MTAB-7251. Mass spectrometry data are deposited on ProteomeXchange with the identifier PXD004640 and PXD011518.

## Supplementary Material

Supplementary Data
