# Supplementary material for: Alternative 3′ UTRs direct localization of functionally diverse protein isoforms in neuronal compartments
Source: Nucleic Acids Res. 2018 Dec 22;47(5):2560–73. doi: 10.1093/nar/gky1270 (PMC6411841; doi:10.1093/nar/gky1270)
Supplement: Supplementary Data [file gky1270_supplemental_files.zip › FigureS1.pdf]

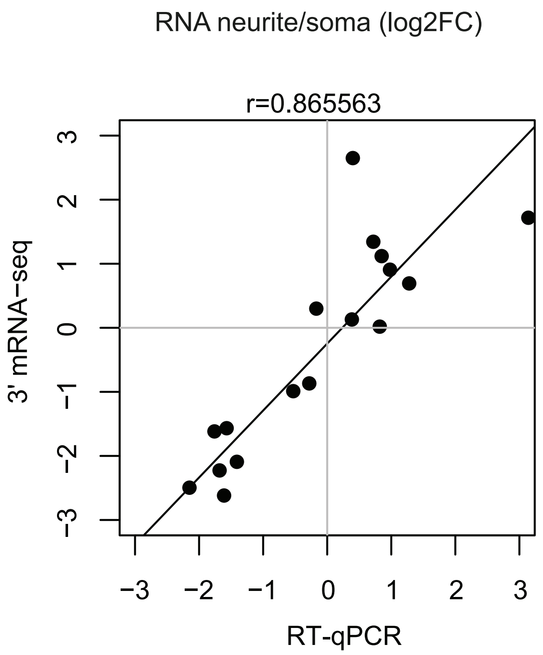

Figure S1, related to Figure 1E. Correaltion between neurite/soma RNA ratios measured by qRT-PCR and 3' mRNA-seq. The plot shows ratio of RNA levels in neurites versus soma (log2 fold change), measured by either RT-qPCR (X) or 3' mRNA-seq (Y). The linear least squares fit between RNAseq and RT-qPCR data is shown. r is the Pearson correlation coefficient.

Figure S1
